# KDM5B decommissions the H3K4 methylation landscape of self-renewal genes during trophoblast stem cell differentiation

**DOI:** 10.1242/bio.031245

**Published:** 2018-05-15

**Authors:** Jian Xu, Benjamin L. Kidder

**Affiliations:** 1 Department of Neurology, Wayne State University School of Medicine, Detroit, MI 48201, USA; 2 Department of Oncology, Wayne State University School of Medicine, Detroit, MI 48201, USA; 3 Karmanos Cancer Institute, Wayne State University School of Medicine, Detroit, MI 48201, USA

**Keywords:** Trophoblast stem cells, Multipotent, Epigenetics, Chromatin, ChIP-Seq, KDM5B, H3K4me3, Differentiation, Histone demethylase

## Abstract

Trophoblast stem (TS) cells derived from the trophectoderm (TE) of mammalian embryos have the ability to self-renew indefinitely or differentiate into fetal lineages of the placenta. Epigenetic control of gene expression plays an instrumental role in dictating the fate of TS cell self-renewal and differentiation. However, the roles of histone demethylases and activating histone modifications such as methylation of histone 3 lysine 4 (H3K4me3/me2) in regulating TS cell expression programs, and in priming the epigenetic landscape for trophoblast differentiation, are largely unknown. Here, we demonstrate that the H3K4 demethylase, KDM5B, regulates the H3K4 methylome and expression landscapes of TS cells. Depletion of KDM5B resulted in downregulation of TS cell self-renewal genes and upregulation of trophoblast-lineage genes, which was accompanied by altered H3K4 methylation. Moreover, we found that KDM5B resets the H3K4 methylation landscape during differentiation in the absence of the external self-renewal signal, FGF4, by removing H3K4 methylation from promoters of self-renewal genes, and of genes whose expression is enriched in TS cells. Altogether, our data indicate an epigenetic role for KDM5B in regulating H3K4 methylation in TS cells and during trophoblast differentiation.

## Abstract (precis)

**Summary:** The histone 3 lysine 4 demethylase KDM5B plays a key role in regulating H3K4 methylation during trophoblast stem cell self-renewal and differentiation. KDM5B regulates the transcriptional profile of TS cells during self-renewal and differentiation, and resets the H3K4 methylation landscape during differentiation by removing H3K4me3 from promoters of self-renewal and TS cell enriched genes.

## INTRODUCTION

The formation of pre-implantation stage blastocysts involves, in part, the segregation of precursor cells that are fated to give rise to embryonic and extraembryonic cell lineages. Cells from the inner cell mass (ICM) of blastocyst-stage embryos are pluripotent and are therefore capable of forming all cells in the adult organism, while murine multipotent trophoblast stem (TS) cells originating from the trophectoderm (TE) layer surrounding the ICM of the blastocyst are capable of differentiating into trophoblast cell types of the placenta. TS cells derived from preimplantation-stage embryos exhibit the ability to self-renew in the presence of external signals including FGF4/heparin and TGFb/Activin supplemented in the culture media or by culturing with mouse embryonic fibroblast (MEF) conditioned media (Kidder, 2014; Tanaka et al., 1998). TS cells are capable of recapitulating placental development *in vitro*, following removal of self-renewal growth factors, by differentiating into extra-embryonic trophoblast fetal tissues of the placenta including trophoblast giant cells, spongiotrophoblasts, glycogen trophoblast cells, and syncytiotrophoblasts. Therefore, TS cells represent an ideal system to investigate the biology of the TE and to model formation of extra-embryonic trophoblast lineage differentiation *in vitro*.

While TS cell and ES cells share the capacity to self-renew indefinitely in the presence of FGF4 and LIF, respectively, they exhibit distinct intrinsic transcriptional networks and differences in patterning of posttranslational modification of histone tails (Rugg-Gunn et al., 2010; Sarmento et al., 2004). However, whether these differences in histone modifications regulate lineage-specific gene expression of TS cells is not fully known. Self-renewal and differentiation of TS cells is dependent on epigenetic control of gene expression, including packaging of DNA into chromatin, and posttranslational modification of histones, which influences the activity of histone modifying enzymes and the expression state of nearby genes. Combined, these actions are important for regulating expression of genes that dictate self-renewal or differentiation.

Methylation of histone 3 lysine 4 (H3K4me3) is predominantly enriched at transcriptional start sites (TSS) of active genes (Barski et al., 2007; Kim et al., 2005; Pokholok et al., 2005; Ringrose and Paro, 2004; Rugg-Gunn et al., 2010; Schubeler et al., 2004). Lysine demethylase 5 (KDM5) members, such as KDM5B, catalyze the demethylation of H3K4me3 (Mosammaparast and Shi, 2010). KDM5 enzymes, such as KDM5B, have traditionally been considered to be transcriptional repressors (Christensen et al., 2007; Klose and Zhang, 2007; Lee et al., 2006), although recent studies have presented a more dynamic role for KDM5B in regulating transcription (He and Kidder, 2017; Kidder et al., 2014; Yamamoto et al., 2014). KDM5B was demonstrated to focus H3K4 methylation near promoters and enhancers by preventing H3K4 methylation from spreading to gene bodies and enhancer shores in ES cells (Kidder et al., 2014). This action may prevent cryptic transcription initiation (Xie et al., 2011). Moreover, KDM5B was shown to regulate RNA polymerase II (RNAPII) occupancy, initiation, and elongation, and alternative splicing in ES cells (He and Kidder, 2017). KDM5B is also important for normal embryonic development (Albert et al., 2013; Catchpole et al., 2011) and ES cell differentiation (Kidder et al., 2013, 2014; Schmitz et al., 2011). While these studies provide insight into the role for KDM5B in regulating H3K4 methylation, transcription and differentiation of ES cells, it is unclear how KDM5B regulates the transcriptional and epigenetic landscapes of TS cells.

Therefore, to investigate the role of KDM5B in regulating H3K4 methylation and gene expression of TS cells and differentiated trophoblast cells, we evaluated genome-wide changes in H3K4 methylation in KDM5B-depleted TS cells and during differentiation. Our findings demonstrate that KDM5B regulates the H3K4 methylome and transcriptome of TS cells and differentiated trophoblast cells, where depletion of KDM5B in TS cells leads to dysregulated expression of TS cell self-renewal genes and H3K4 methylation. We also demonstrate that KDM5B resets the H3K4 methylation landscape during differentiation by demethylating self-renewal and TSC-enriched genes. These findings implicate an epigenetic role for KDM5B in regulating H3K4 methylation in TS cells and during trophoblast differentiation.

## RESULTS

### Depletion of KDM5B leads to an altered transcriptome of TS cells

**Fig. 1.**
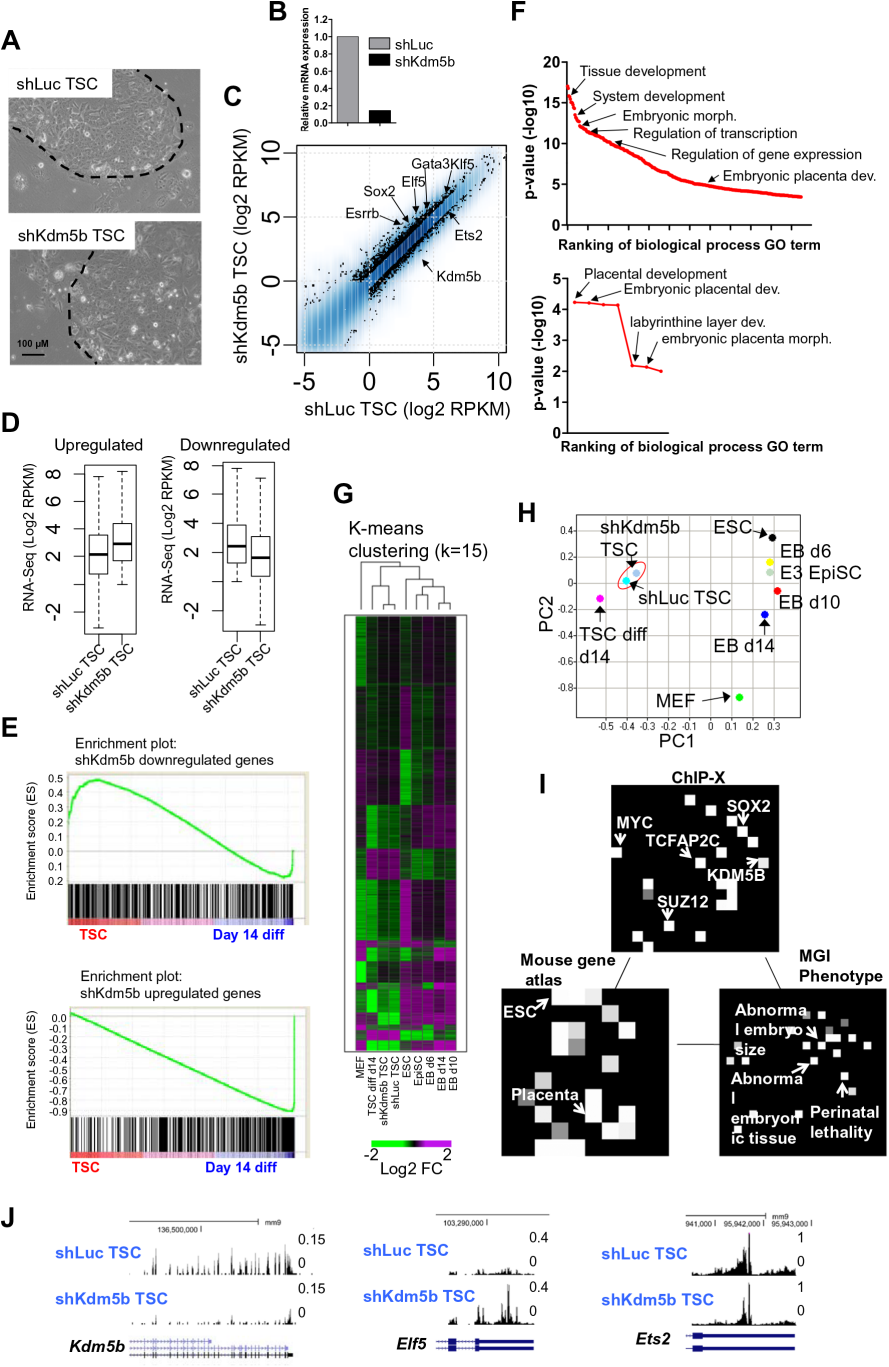
**KDM5B regulates expression of self-renewal genes in TS cells.** (A) TS cells transduced with shLuc (control) or shKdm5b lentiviral particles and stably selected with puromycin. Dotted lines outline boundary of TS cell colony. Representative micrographs from at least three independent experiments are shown. (B) Relative RNA-Seq expression level of *Kdm5b* in shLuc and shKdm5b TS cells. *Kdm5b* RNA-Seq mRNA levels (RPKM) were normalized to shLuc TS cells. (C) Scatter plot of RNA-Seq gene expression analysis between shLuc and shKdm5b TS cells. Log2 adjusted differentially expressed genes are plotted (>twofold, RPKM>3). At least two biological replicates were performed for RNA-Seq analyses. (D) Boxplot of RNA-Seq data: upregulated and downregulated genes in shLuc and shKdm5b TS cells (log2 RPKM). (E) Gene set enrichment analysis (GSEA) plot of downregulated (top) and upregulated (bottom) differentially expressed genes in KDM5B-depleted TS cells relative to shLuc TS cells. Note that the expression of the majority of genes downregulated in shKdm5b TS cells is enriched in undifferentiated TS cells (top plot), while expression of genes that are upregulated in shKdm5b TS cells is enriched in differentiated TS cells (bottom plot). A positive enrichment score indicates that expression of genes is enriched in undifferentiated TS cells, while a negative enrichment score indicates that expression of genes is enriched in differentiated TS cells. (F) DAVID gene ontology (GO) functional annotation of differentially expressed genes between shLuc and shKdm5b TS cells. The bottom graph shows significantly enriched placental and trophoblast GO terms. (G) K-means clustering analysis of RNA-Seq data. Differentially expressed genes (>twofold) clustered according to k-means. (H) Principal component analysis (PCA) of RNA-Seq expression between shLuc and shKdm5b TS cells, day 14 differentiated TS cells, ES cells, day 6, 10, and 14 embryoid body (EB) differentiated ES cells, EpiSCs, and MEFs. (I) Network2Canvas (N2C) (Tan et al., 2013) analyses of differentially expressed genes between shLuc and shKdm5b TS cells. In each canvas, each node (square) represents a gene list (shLuc versus Kdm5b DE genes in TS cells) associated with a functional term in a gene-set library (ChIP-X, mouse gene atlas, and MGI phenotype). The terms are organized on the canvas based on the similarity of their gene-set content. The brightness (white) of each node is determined by its *P* value. (J) Custom tracks of RNA-Seq data in the UCSC genome browser.

To study the function of KDM5B in TS cells, we knocked down *Kdm5b* using lentiviral particles encoding short hairpin RNAs (shRNA) (see the Materials and Methods). shKdm5b-shRNA-1 resulted in the greatest mRNA knockdown of Kdm5b in ES cells (Kidder et al., 2013, 2014), and was therefore used for this study. shKdm5b TS cells and control Luciferase-shRNA (shLuc) TS cells were stably selected in the presence of 1 µg/ml puromycin (Fig. 1A). Notably, depletion of KDM5B did not result in a significantly altered TS cell colony morphology (Fig. 1A). Depletion of Kdm5b in TS cells resulted in an 86% reduction of *Kdm5b* mRNA as evaluated using RNA-Seq (Fig. 1B). A comparison of global expression profiles using RNA-Seq identified 2631 differentially expressed genes between control (shLuc) and shKdm5b TS cells, including 1468 genes whose expression was upregulated and 893 genes whose expression was downregulated at least twofold in shKdm5b TS cells. Interestingly, we found that transcription factors (TF) involved in TS cell self-renewal, including Elf5, Gata3, Klf5, Esrrb, and Sox2 were upregulated in shKdm5b TS cells, while Ets2 was downregulated in shKdm5b TS cells (Fig. 1C). Boxplots revealed that the expression level of genes that were upregulated in shKdm5b TS cells was slightly lower in shLuc TS cells relative to genes that were downregulated in shKdm5b TS cells (Fig. 1D). These results suggest that depletion of KDM5B leads to decreased expression of TSC-enriched genes and increased expression of trophoblast-lineage specific genes. In support of this model, a comparison of these differentially expressed (DE) genes with global expression data from undifferentiated TS cells and day 14 differentiated TS cells, using gene set enrichment analysis (GSEA) (Subramanian et al., 2005), showed that downregulated genes in shKdm5b TS cells are enriched in undifferentiated TS cells while upregulated genes are enriched in differentiated TS cells (Fig. 1E). These results suggest that KDM5B regulates expression of TSC-enriched genes during self-renewal. In addition, DAVID (Dennis et al., 2003) gene ontology (GO) terms enriched in DE genes include tissue development, system development, embryonic morphogenesis, regulation of transcription, and embryonic placental development (Fig. 1F). Additional statistically significant GO terms enriched in DE genes include placental development, labyrinthine layer development, and embryonic placental morphogenesis.

To further evaluate the transcriptional profile of shKdm5b TS cells, hierarchical and K-means clustering analysis was used to identify major patterns of expression variability between shLuc and shKdm5b TS cells, day 14 differentiated TS cells, ES cells, epiblast stem cells (EpiSCs), day 6, 10, and 14 embryoid body (EB) differentiated ES cells (Fig. 1G). These results demonstrate that the transcriptome of shKdm5b TS cells is more similar to shLuc TS cells relative to differentiated TS cells and other stem cells derived from preimplantation-stage embryos including ES cells and EpiSCs, and differentiated ES cells. The dimensionality reduction tool, Principal component analysis (PCA), confirmed these findings: we found that the two-dimensional spatial proximity of shKdm5b TS cells was closer to shLuc TS cells relative to differentiated TS cells or other undifferentiated or differentiated stem cells derived from preimplantation stage embryos (Fig. 1H). These results are in alignment with findings we observed previously for shKdm5b ES cells. While depletion of KDM5B in ES cells resulted in a slight decrease in self-renewal genes and dysregulated expression of several genes implicated in ES cell self-renewal, loss of KDM5B did not result in a collapse of the self-renewal transcriptional network (Kidder et al., 2013). Further annotation of the DE genes in shKdm5b TS cells using Network2canvas (Tan et al., 2013) revealed binding sites of TFAP2C, KDM5B, MYC, and SUZ12 (Fig. 1I, top), expression in placenta and ES cells (Fig. 1I, bottom left), and involvement in abnormal embryo size, embryonic tissue, and placental lethality (Fig. 1I, bottom right). Visualization of custom tracks on the UCSC genome browser revealed downregulation of genes such as *Kdm5b* and *Ets2*, and upregulation of *Elf5* in shKdm5b TS cells (Fig. 1J). Overall, these results suggest that while depletion of KDM5B leads to altered expression of self-renewal and TSC-enriched genes, it does not lead to a collapse of the self-renewal network.

### KDM5B regulates the H3K4 methylation landscape of TS cells

**Fig. 2.**
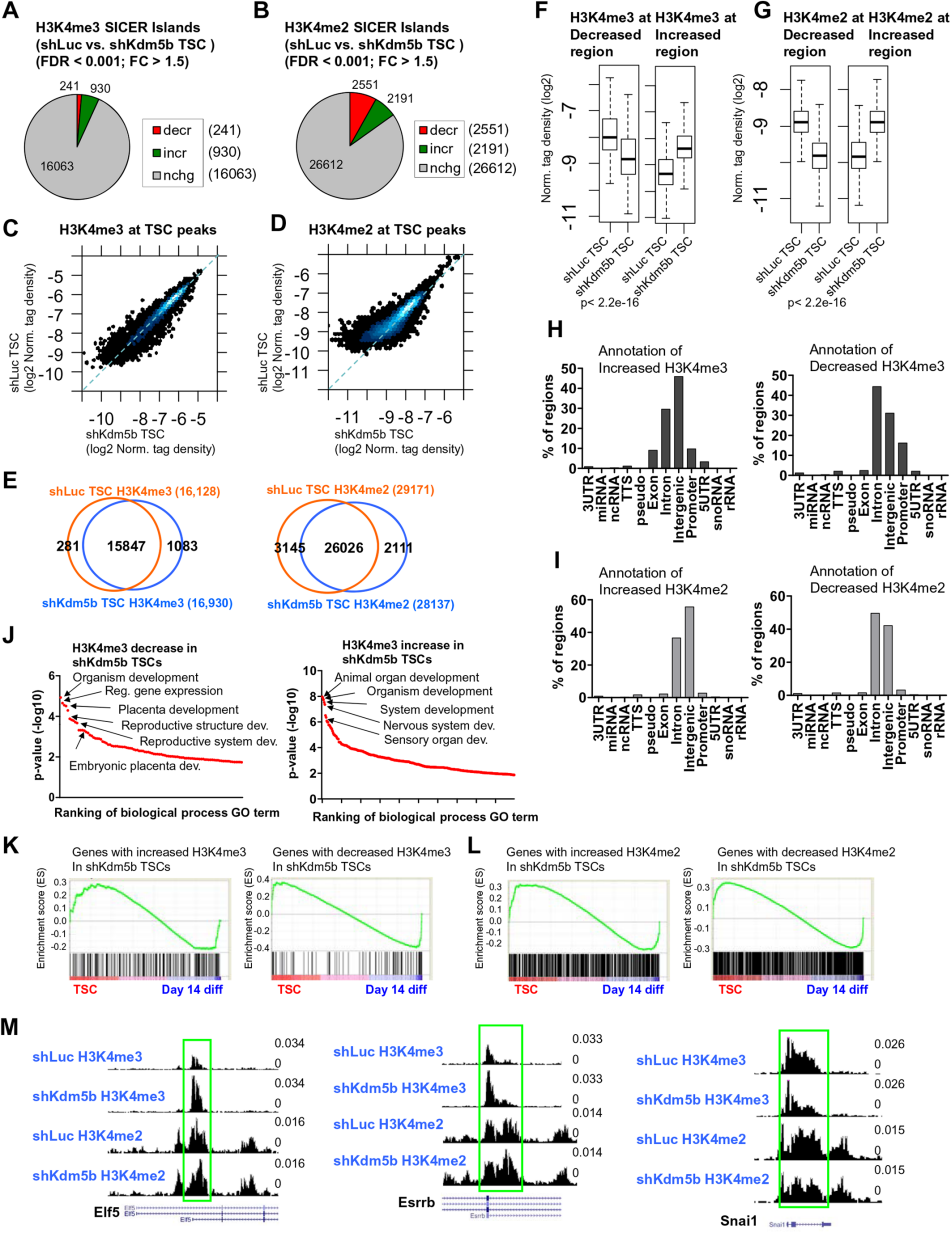
**KDM5B regulates the H3K4 methylation landscape of TS cells.** Depletion of KDM5B leads to altered (A) H3K4me3 and (B) H3K4me2 levels in TS cells (See the Materials and Methods section for SICER-analysis; fold-change >1.5, FDR <0.001). Scatter plots of (C) H3K4me3 and (D) H3K4me2 densities at TSC ChIP-enriched peaks (log2 normalized tag density). (E) Venn diagrams showing overlap of H3K4me3 (left) and H3K4me2 (right) in shLuc and shKdm5b TS cells at SICER-defined islands. (F,G) Boxplots of (F) H3K4me3 and (G) H3K4me2 densities at regions of increased or decreased H3K4me3 or H3K4me2 in shKdm5b TS cells relative to shLuc TS cells. (H,I) Annotation of regions with increased or decreased (H) H3K4me3 or (I) H3K4me2 in KDM5B-depleted TS cells using HOMER software. (J) DAVID gene ontology (GO) functional annotation of genes with altered H3K4me3 in KDM5B-depleted TS cells relative to shLuc TS cells. (K,L) GSEA of genes with (K) increased (left) or decreased (right) H3K4me3 or (L) H3K4me2 in shKdm5b TS cells relative to shLuc TS cells. (M) Custom views of ChIP-Seq data in the UCSC genome browser.

To evaluate the genome-wide distribution of H3K4 methylation in KDM5B-depleted TS cells we performed chromatin immunoprecipitation followed by next-generation sequencing (ChIP-Seq). Results from these experiments revealed altered levels of H3K4me3 (Fig. 2A) and H3K4me2 (Fig. 2B) at a number of regions in shKdm5b TS cells [SICER (Zang et al., 2009) islands; FDR <0.001, fold-change >1.5; see the Materials and Methods]. Interestingly, we observed a greater number of regions with increased H3K4me3 relative to regions with decreased H3K4me3 in shKdm5b TS cells relative to shLuc TS cells (Fig. 2A). However, we observed a relatively equal number of regions with increased and decreased H3K4me2 in shKdm5b TS cells (Fig. 2B). Scatter plots confirmed these changes in H3K4me3 (Fig. 2C) and H3K4me2 (Fig. 2D) levels in shKdm5b TS cells. Venn diagrams revealed that shKdm5b TS cells exhibited an increased number of H3K4me3 regions and a relatively equal distribution of increased and decreased H3K4me2 regions (Fig. 2E). Moreover, boxplots revealed that regions with high enrichment of H3K4me3 in shLuc TS cells exhibited decreased H3K4me3 levels in shKdm5b TS cells, while regions with moderate enrichment of H3K4me3 in shLuc TS cells exhibited increased H3K4me3 levels in shKdm5b TS cells (Fig. 2F). Likewise, regions with high enrichment of H3K4me2 in shLuc TS cells exhibited decreased H3K4me2 levels in shKdm5b TS cells, while regions with moderate enrichment of H3K4me2 in shLuc TS cells exhibited increased H3K4me2 levels in shKdm5b TS cells (Fig. 2G). These results suggest that KDM5B is important for maintaining elevated H3K4me3 and H3K4me2 levels at a subset of TSC-enriched genes, and for dampening H3K4me3 and H3K4me2 levels at a different subset of genes.

Annotation of regions using homer software (Heinz et al., 2010) of increased H3K4me3 in shKdm5b TS cells showed that they are mainly located at intergenic, intron, promoter, and exon regions (Fig. 2H, left), while decreased H3K4me3 regions are located at intron, intergenic, and promoter regions (Fig. 2H, right). In addition, annotation of increased and decreased H3K4me2 regions showed that they are predominantly located in intron and intergenic regions (Fig. 2I). DAVID (Dennis et al., 2003) was subsequently used to functionally annotate regions of differential H3K4 methylation in shKdm5 and shLuc TS cells. Our findings show that several gene ontology terms, including organism development, regulation of gene expression, placental development, reproductive structure development, and reproductive system development were overrepresented in genes with decreased H3K4me3 (Fig. 2J, left). In addition, developmental GO terms were enriched in genes that displayed increased H3K4me3 in shKdm5b TS cells (Fig. 2J, right). To further understand the expression state of genes with altered H3K4me3 or H3K4me2 in KDM5B-depleted TS cells, we compared these genes to expression data from undifferentiated TS cells and day 14 differentiated TS cells using gene set enrichment analysis (GSEA) (Subramanian et al., 2005). These results show that genes with increased or decreased H3K4me3 are relatively evenly distributed between being expressed in undifferentiated TS cells or in differentiated TS cells (Fig. 2K-L). Increased H3K4me3 is visible at several self-renewal genes (*Elf5*, *Esrrb*) in custom UCSC genome browser views (Fig. 2M). Moreover, decreased H3K4me3 is also visible in a genome browser view (Fig. 2M, right).

### KDM5B removes H3K4 methylation at TS cell enriched genes during differentiation

**Fig. 3.**
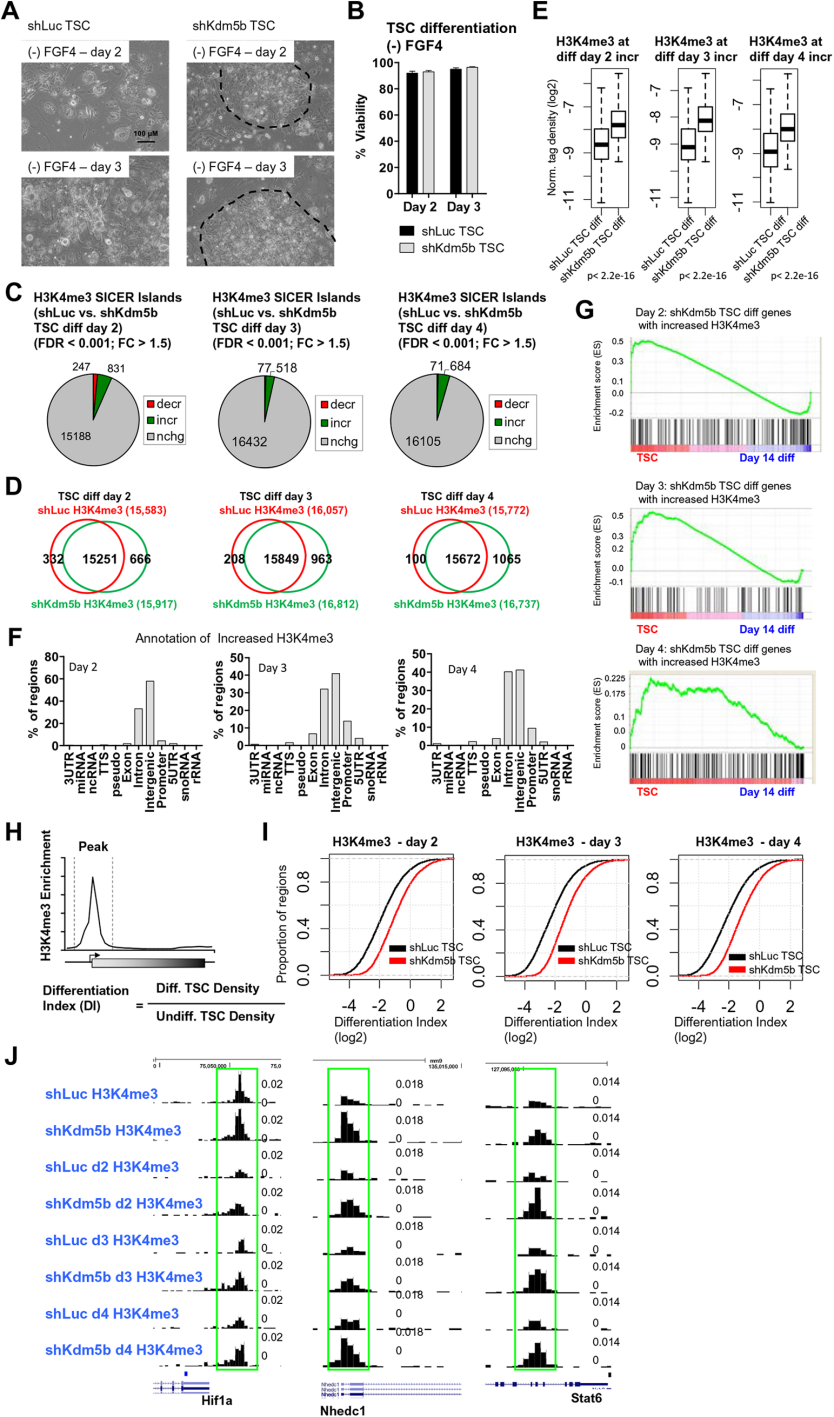
**KDM5B demethylates H3K4me3 at TSC-enriched genes during early differentiation.** (A) Bright-field microscopy of day 2–3 differentiated shLuc and shKdm5b TS cells. (B) Percent viability of day 2–3 differentiated shLuc and shKdm5b TS cells. (C) Differentiated KDM5B-depleted TS cells exhibit elevated H3K4me3 ChIP-Seq levels relative to differentiated shLuc TS cells (See the Materials and Methods section for SICER-analysis; fold-change >1.5, FDR <0.001). (D) Venn diagrams showing overlap of H3K4me3 in day 2–4 differentiated shLuc and shKdm5b TS cells at SICER-defined islands. (E) Boxplots of H3K4me3 densities at regions with increased H3K4me3 in day 2, day 3, and day 4 differentiated shKdm5b TS cells. (F) Annotation of regions with increased H3K4me3 in shKdm5b versus shLuc day 2, day 3, or day 4 differentiated TS cells using HOMER software. (G) GSEA of genes with increased H3K4me3 in shKdm5b versus shLuc day 2, day 3, or day 4 differentiated TS cells. (H) Schematic describing the calculation used to determine the differentiation index (DI) at H3K4me3 marked genes in ES cells. The DI is calculated from the ratio of the density of H3K4me3 in differentiated TS cells to the density of H3K4me3 in undifferentiated TS cells. (I) Empirical cumulative distribution for the DI of H3K4me3 across genes marked by H3K4me3 in undifferentiated TS cells for shLuc (black) and shKdm5b (red) TS cells. Y-axis shows the percentage of regions that exhibit a DI less than the value specified by the x-axis. A line shifted to the right means a systematic increase in the differentiation index. *P*-value for all <2.2E-16 (Kolmogorov–Smirnov test). Note the decreased DI for genes marked by H3K4me3 in shKdm5b TS cells. (J) Custom views of ChIP-Seq data in the UCSC genome browser.

To investigate whether KDM5B regulates the H3K4 methylation landscape during early differentiation of TS cells we cultured shLuc and shKdm5b TS cells in the absence of FGF4 over a time-course of 2–4 days to induce differentiation. While shLuc TS cell colonies lost their tight cell-cell contact at the colony periphery, and became scattered, after two days of differentiation (Fig. 3A, left), day 2 and day 3 differentiated shKdm5b TS cell colonies maintained a TSC-like morphology (Fig. 3A, right), suggesting that depletion of KDM5B leads to delayed differentiation. Also, we did not observe altered viability of differentiated shKdm5b TS cells relative to differentiated shLuc TS cells (day 2, *P*=0.1; day 3, *P*=0.17) (Fig. 3B).

Next, we surveyed the genome-wide distribution of H3K4me3 in day 2, day 3, and day 4 differentiated shLuc and shKdm5b TS cells using ChIP-Seq. Our findings show that depletion of KDM5B in TS cells leads to increased H3K4me3 levels during early differentiation (day 2–4) relative to control (shLuc) TS cells [SICER (Zang et al., 2009) islands; FDR <0.001, fold-change >1.5; see the Materials and Methods] (Fig. 3C). We also observed a sequential increase in the number of H3K4me3 ChIP-enriched peaks in shKdm5b TS cells during differentiation (Fig. 3D). We also evaluated H3K4me3 densities at regions of increased H3K4me3 in day 2, day 3, and day 4 differentiated shKdm5b TS cells relative to shLuc TS cells. Box plots reveal that day 2, day 3, and day 4 differentiated KDM5B-depleted TS cells exhibited increased H3K4me3 ChIP-Seq levels relative to differentiated shLuc TS cells (Fig. 3E). Annotation of regions with increased H3K4me3 during early differentiation of KDM5B-knockdown TS cells revealed that they are located in intron, promoter, intergenic, and exon regions (Fig. 3F).

We then analyzed the expression state of genes with increased H3K4me3 in day 2, day 3, and day 4 differentiated shKdm5b TS cells by comparing these genes with expression data from undifferentiated TS cells and day 14 differentiated TS cells using GSEA. Our results show that expression of genes with increased H3K4me3 in differentiated shKdm5b TS cells (day 2, day 3, day 4) is mainly enriched in undifferentiated TS cells (Fig. 3G). These results suggest that TSC-enriched genes are not fully demethylated during differentiation in the absence of KDM5B, demonstrating an important role for KDM5B in removing H3K4me3 at TSC-enriched genes during differentiation.

To further explore the relationship between depletion of KDM5B and regulation of H3K4me3 at TSC-enriched genes during differentiation, we quantified the relative ratio of H3K4me3 density in day 2–4 differentiated TS cells to that in undifferentiated TS cells, which we have termed the differentiation index (DI) (Fig. 3H). Using this calculation, we observed a decrease in the DI for H3K4me3 in day 2–4 differentiated shLuc TS cells relative to day 2–4 differentiated KDM5B-depleted TS cells (Fig. 3I). These results show that depletion of KDM5B regulates H3K4 demethylation during TS cell differentiation. Increased H3K4me3 is visible at several self-renewal and trophoblast genes (*Hif1a*, *Stat6*, *Nhedc1/Slc9b1*) in custom UCSC genome browser views (Fig. 3J).

**Fig. 4.**
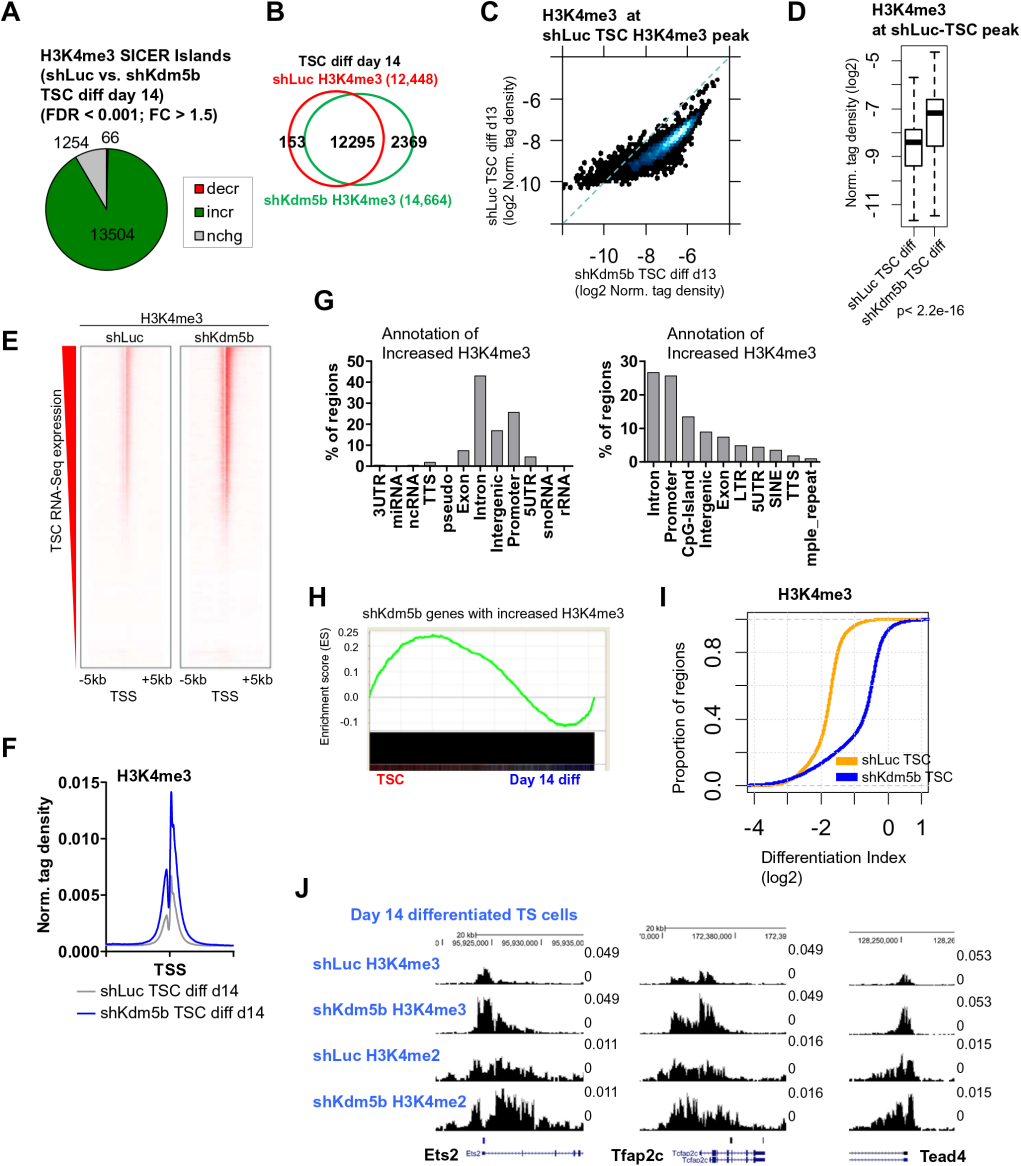
**KDM5B demethylates H3K4me3 at TSC-enriched genes during differentiation.** (A) Differentiated KDM5B-depleted TS cells exhibit elevated H3K4me3 ChIP-Seq levels relative to differentiated shLuc TS cells (See the Materials and Methods section for SICER-analysis; fold-change >1.5, FDR <0.001). (B) Venn diagrams showing overlap of H3K4me3 in day 14 differentiated shLuc and shKdm5b TS cells at SICER-defined islands. (C) Scatter plot of H3K4me3 densities at TSC ChIP-enriched peaks (log2 normalized tag density). (D) Boxplot of H3K4me3 density at TSC-defined ChIP-Seq peaks in shLuc and shKdm5b day 14 differentiated TS cells. (E) Heat maps of H3K4me3 densities at transcriptional start sites (TSS) in shLuc and shKdm5b day 14 differentiated TS cells (genes were sorted by their expression in undifferentiated TS cells). (F) Average profile of H3K4me3 in shLuc and shKdm5b day 14 differentiated TS cells. (G) Annotation of regions with increased H3K4me3 in shKdm5b versus shLuc day 14 differentiated TS cells using HOMER software. (H) GSEA of genes with increased H3K4me3 in shKdm5b versus shLuc day 14 differentiated TS cells. (I) Empirical cumulative distribution for the differentiation index (DI) of H3K4me3 across genes marked by H3K4me3 in undifferentiated TS cells for shLuc (orange) and shKdm5b (blue) TS cells. Y-axis shows the percentage of regions that exhibit a DI less than the value specified by the x-axis. A line shifted to the right means a systematic increase in the differentiation index. *P*-value for all <2.2E-16 (Kolmogorov–Smirnov test). Note the decreased DI for genes marked by H3K4me3 in shKdm5b TS cells. (J) Custom views of ChIP-Seq data in the UCSC genome browser.

We also investigated whether KDM5B regulates the global profile of H3K4 methylation following two weeks of TSC differentiation. To this end, we surveyed the genome-wide distribution of H3K4me3 in day 14 differentiated shLuc and shKdm5b TS cells using ChIP-Seq. Results from these experiments showed that KDM5B-depleted TS cells exhibited globally increased H3K4me3 levels relative to control (shLuc) TS cells [SICER (Zang et al., 2009) islands; FDR <0.001, fold-change >1.5; see the Materials and Methods] (Fig. 4A). We also observed an increase in the number of H3K4me3 ChIP-enriched peaks in shKdm5b TS cells (Fig. 4B). We then evaluated H3K4me3 densities at shluc-TSC-H3K4me3 ChIP-enriched peaks. A scatter plot (Fig. 4C) and box plot (Fig. 4D) showed that day 14 differentiated KDM5B-depleted TS cells exhibited increased H3K4me3 ChIP-Seq levels relative to differentiated shLuc TS cells. Moreover, heat maps (Fig. 4E) and average profiles (Fig. 4F) of H3K4me3 densities around transcriptional start sites (TSS) further revealed increased H3K4me3 levels in differentiated shKdm5b TS cells relative to differentiated shLuc TS cells. Annotation of differentially methylated regions showed that regions with increased H3K4me3 in KDM5B-depleted day 14 differentiated TS cells are located in intron, promoter, intergenic, and exon regions (Fig. 4G, left). Further annotation also revealed that regions of increased H3K4me3 in day 14 differentiated shKdm5b TS cells are enriched with CpG-islands (Fig. 4G, right), suggesting that KDM5B may be important for removing H3K4me3 at CpG-islands during differentiation.

Next, we analyzed the expression state of genes with increased H3K4me3 in differentiated shKdm5b TS cells by comparing these genes with expression data from undifferentiated TS cells and day 14 differentiated TS cells using GSEA as described above. These results show that the expression of the majority of genes with increased H3K4me3 in differentiated shKdm5b TS cells is enriched in undifferentiated TS cells (Fig. 4H). Together, these results show that in the absence of KDM5B, H3K4me3 is not fully demethylated at TSC-enriched genes during differentiation, suggesting a critical role for KDM5B in removing H3K4me3 at TSC-enriched genes during differentiation.

We then explored the relationship between depletion of KDM5B and regulation of H3K4me3 at TSC-enriched genes during differentiation by quantifying the relative ratio of H3K4me3 density in differentiated TS cells (day 14) to that in undifferentiated TS cells using the DI, as described in Fig. 3H. Using this calculation, we observed a decrease in the DI for H3K4me3 in shLuc TS cells relative to KDM5B-depleted TS cells (Fig. 4I). These results show that depletion of KDM5B impacts demethylation of H3K4me3 during TS cell differentiation. Increased H3K4me3 is visible at several self-renewal genes (*Ets2*, *Tfap2c*, *Tead4*) in custom UCSC genome browser views (Fig. 4J).

**Fig. 5.**
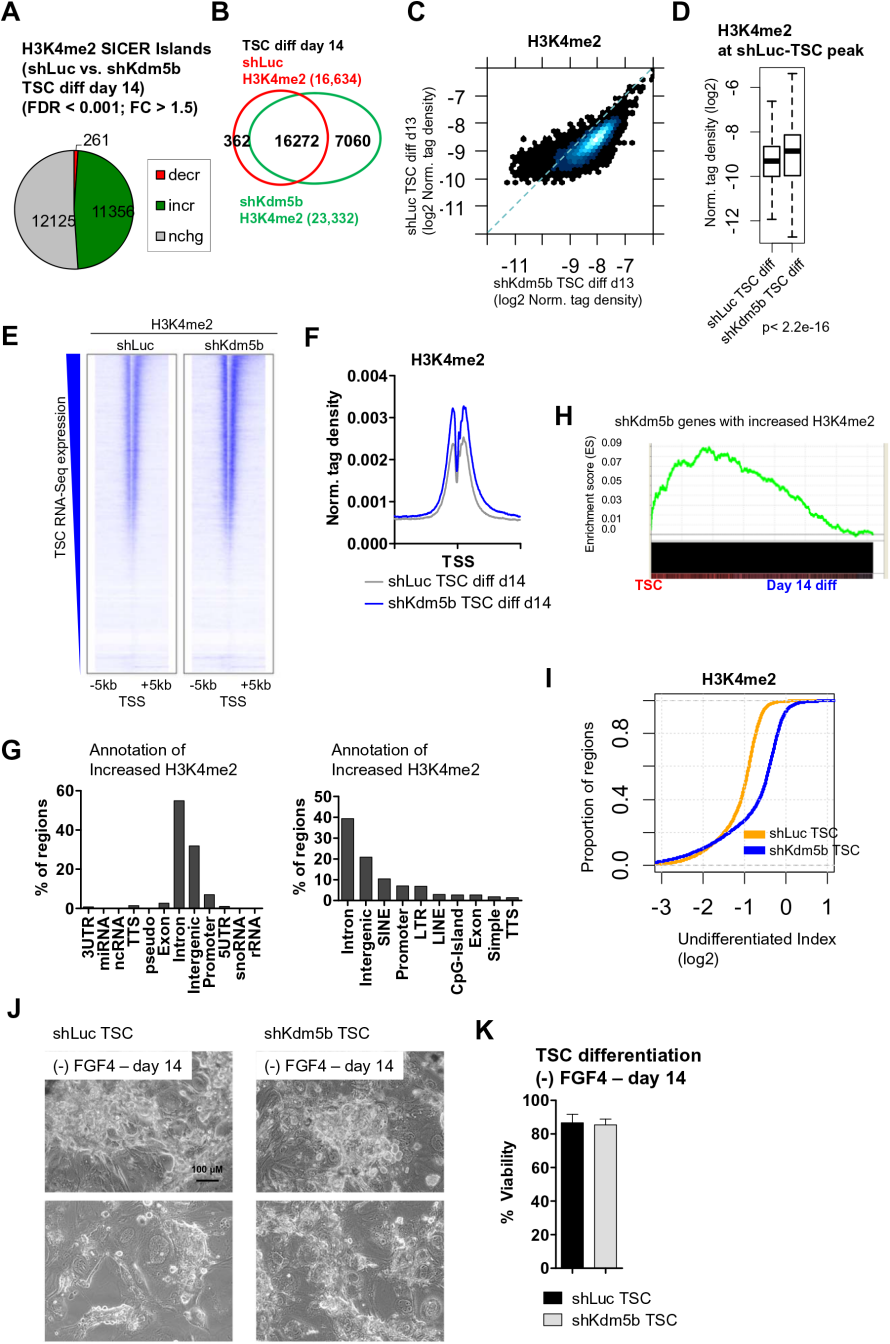
**KDM5B demethylates H3K4me2 at TSC-enriched genes during differentiation.** (A) Differentiated KDM5B-depleted TS cells exhibit elevated H3K4me2 ChIP-Seq levels relative to differentiated shLuc TS cells (See the Materials and Methods section for SICER-analysis; fold-change >1.5, FDR <0.001). (B) Venn diagrams showing overlap of H3K4me2 in day 14 differentiated shLuc and shKdm5b TS cells at SICER-defined islands. (C) Scatter plot of H3K4me2 densities at TSC ChIP-enriched peaks (log2 normalized tag density). (D) Boxplot of H3K4me2 density at TSC-defined ChIP-Seq peaks in shLuc and shKdm5b day 14 differentiated TS cells. (E) Heat maps of H3K4me2 densities at transcriptional start sites (TSS) in shLuc and shKdm5b day 14 differentiated TS cells (genes were sorted by their expression in undifferentiated TS cells). (F) Average profile of H3K4me2 in shLuc and shKdm5b day 14 differentiated TS cells. (G) Annotation of regions with increased H3K4me2 in shKdm5b versus shLuc day 14 differentiated TS cells using HOMER software. (H) GSEA of genes with increased H3K4me2 in shKdm5b versus shLuc day 14 differentiated TS cells. (I) Empirical cumulative distribution for the differentiation index (DI) of H3K4me2 across regions marked by H3K4me2 in undifferentiated TS cells for shLuc (orange) and shKdm5b (blue) TS cells. Y-axis shows the percentage of genes that exhibit a DI less than the value specified by the x-axis. A line shifted to the right means a systematic increase in the differentiation index. *P*-value for all <2.2E-16 (Kolmogorov–Smirnov test). Note the decreased DI for genes marked by H3K4me2 in shKdm5b TS cells. (J) Bright-field microscopy of day 14 differentiated shLuc and shKdm5b TS cells. (K) Percent viability of day 14 differentiated shLuc and shKdm5b TS cells.

We also investigated whether KDM5B regulates H3K4me2 during TS cell differentiation by performing ChIP-Seq using day 14 differentiated shLuc and shKdm5b TS cells in a similar way as described above. These results show that KDM5B-depleted TS cells display global increases in H3K4me2 ChIP-Seq levels relative to shLuc TS cells (SICER islands; FDR <0.001, fold-change >1.5; see the Materials and Methods) (Fig. 5A). In addition, we observed a greater number of H3K4me2 ChIP-enriched peaks in differentiated shKdm5b TS cells relative to differentiated shLuc TS cells (Fig. 5B), and an overall increase in H3K4me2 densities at shluc-TSC-H3K4me2 ChIP-enriched peaks, as evaluated using a scatter plot (Fig. 5C) and box plot (Fig. 5D). Heat maps (Fig. 5E) and average profiles (Fig. 5F) of H3K4me2 densities around transcriptional start sites (TSS) also revealed increased H3K4me2 in differentiated KDM5B-depleted TS cells. Annotation of regions with differential H3K4me2 showed that regions with increased H3K4me2 in differentiated KDM5B-depleted TS cells are located in intron, intergenic, and promoter regions (Fig. 5G, left). Further annotation also revealed that regions of increased H3K4me2 are enriched with SINE repetitive DNA elements (Fig. 5G, right).

We also analyzed the expression state of genes with increased H3K4me2 in day 14 differentiated shKdm5b TS cells by comparing these genes with expression data from undifferentiated TS cells and day 14 differentiated TS cells using GSEA. Our results demonstrate that the expression of genes with increased H3K4me2 in differentiated KDM5B-depleted TS cells is enriched in undifferentiated TS cells (Fig. 5H). Overall, these results show that depletion of KDM5B leads to incomplete demethylation of H3K4me2 at TS cell self-renewal genes during differentiation, further demonstrating that KDM5B demethylates TSC-enriched genes during differentiation.

We further explored the relationship between depletion of KDM5B and regulation of H3K4me2 at TSC-enriched genes during differentiation by quantifying the relative ratio of H3K4me2 density in differentiated TS cells (day 14) to that in undifferentiated TS cells (differentiation index; DI). Using this calculation, we observed a decrease in the DI for H3K4me2 in shLuc TS cells relative to KDM5B-depleted TS cells (Fig. 5I). While depletion of KDM5B resulted in delayed H3K4 demethylation and delayed differentiation in the FGF4, depletion of KDM5B did not completely block differentiation (Fig. 5J). Moreover, we did not observe altered viability of day 14 differentiated shKdm5b TS cells relative to shLuc TS cells (*P*=0.44) (Fig. 5J–K). Altogether, these results suggest that depletion of KDM5B leads to a delay in demethylation of H3K4me3 and H3K4me2 during TS cell differentiation.

## DISCUSSION

Here, we describe an epigenetic role for KDM5B in regulating the H3K4 methylomes and transcriptomes of undifferentiated and differentiated TS cells. While H3K4 methylation is known to be localized at active promoters (Barski et al., 2007), it is unclear how H3K4 methylation is regulated in TS cells and during trophoblast-lineage differentiation. In particular, what is the role for the KDM5B demethylase in regulating H3K4me3 in TS cells and during differentiation? By examining the epigenetic and transcriptional landscapes of KDM5B-depleted TS cells, we found that loss of KDM5B leads to dysregulated expression of genes whose expression is enriched in TS cells, including genes implicated in TS cell self-renewal. However, we did not observe a full collapse of the self-renewal transcriptional network in KDM5B-depleted TS cells, where an evaluation of RNA-Seq data using hierarchical clustering analysis demonstrated that shKdm5b TS cells clustered closer to shLuc TS cells relative to differentiated TS cells, stem cells derived from preimplantation-stage embryos (ES cells and EpiSCs), and differentiated MEFs and EBs (Fig. 1G). These results are consistent with previous findings which demonstrated that while depletion of KDM5B in ES cells leads to dysregulated gene expression, it does not lead to a complete collapse of the self-renewal transcriptional network (Kidder et al., 2013). Interestingly, while we observed downregulation of TS cell enriched genes in shKdm5b TS cells, and upregulation of genes whose expression is enriched in differentiated TS cells, several self-renewal genes such as *Esrrb*, *Sox2*, *Elf5*, *Gata3*, and *Klf5* were upregulated in shKdm5b TS cells. One possible explanation for these findings is that increased expression of transcription factors (TFs) implicated in TS cell self-renewal may attenuate the expression of other TSC-enriched genes. In this case, upregulated expression of self-renewal TFs, several of which co-occupy many target genes (Kidder and Palmer, 2010), may lead to dysregulated expression of downstream targets. This phenomena has been observed in ES cells, where overexpression of OCT4 leads to differentiation of ES cells (Niwa et al., 2000). We propose that KDM5B acts in part to dampen the transcriptional output of key self-renewal genes in TS cells by regulating the H3K4 methylation landscape. In support of this model, our results show that depletion of KDM5B in TS cells resulted in increased H3K4me3 at self-renewal genes (Fig. 2M), which was accompanied by increased expression (Fig. 1C).

We also propose that KDM5B is a key epigenetic regulator of the H3K4 methylome during TS cell differentiation, where KDM5B acts to reset the epigenetic landscape during differentiation by demethylating H3K4 at self-renewal genes and at genes whose expression is enriched in TS cells. In support of this model, we observed maintenance of H3K4 methylation (H3K4me3, H3K4me2) at self-renewal genes in differentiated shKdm5b TS cells relative to differentiated shLuc TS cells (Figs 3J and 4J). We also established a metric to quantify the relative differentiation of KDM5B-depleted TS cells by surveying H3K4me3/H3K4me2 densities in undifferentiated versus differentiated TS cells. Results from these analyses demonstrate that KDM5B-depleted TS cells exhibit incomplete demethylation of TSC-enriched genes during differentiation. These findings suggest that KDM5B plays a critical role in deactivating H3K4 methylation at self-renewal genes and at genes whose expression is enriched in TS cells to reset H3K4 methylation.

Self-renewal of ES cells and TS cells is dependent on extrinsic signals and intrinsic epigenetic and transcriptional networks. While TS cells share similar characteristics as ES cells (Kidder and Palmer, 2010), lineage-specific differences exist in epigenetic pathways between these cell types. In particular, ES cells contain bivalent chromatin domains consisting of the activating H3K4me3 and repressive H3K27me3 histone modifications, which are enriched at developmental genes that are repressed in ES cells but active during differentiation. However, H3K4me3/H3K27me3 bivalent domains are largely absent in TS cells (Rugg-Gunn et al., 2010). While KDM5B was previously found to regulate H3K4me3 at H3K4me3/H3K27me3 bivalently marked genes during differentiation of ES cells (Kidder et al., 2014), because TS cells exhibit a limited overlap between these two modifications, the role for KDM5B in regulating TS cell differentiation is likely independent of a mechanism involving regulation of these bivalent domains.

In summary, we describe a mechanism whereby KDM5B regulates H3K4 methylation on a global scale in TS cells to regulate expression of self-renewal genes and to reset the H3K4 methylome during trophoblast differentiation. These results provide novel insight into the epigenetic landscape that supports self-renewal and differentiation of TS cells.

## MATERIALS AND METHODS

### TS cell culture

TS cells were cultured as previously described with minor modifications (Kidder and Palmer, 2010, 2012). Briefly, TSC-BK12 TS cells (Kidder and Palmer, 2010) were cultured on irradiated MEFs in RPMI 1640 medium supplemented with 20% FBS (Gibco), 1 mM sodium pyruvate (Gibco), 100 µM β-mercaptoethanol (EMD Millipore, Billerica, USA), 2 mM L-glutamine (Gibco), 25 ng/ml recombinant human FGF4 (1X) (R&D Systems, Minneapolis, USA), and 1 µg/ml heparin (1X) (Sigma-Aldrich). TS cells have been tested for mycoplasma using a kit from Thermo Fisher Scientific (MycoFluor Mycoplasma Detection Kit). For ChIP experiments, TS cells were transitioned to dishes containing TS cell medium, 70% iMEF-conditioned medium, and 1.5X FGF4/heparin, and cultured at 37°C with 5% CO_2_. TS cells were passed at least three times to remove iMEFs by washing with PBS, and dissociated with trypsin using serological pipettes (sc-200279, sc-200281). For differentiation experiments, TS cells were cultured in feeder-free dishes containing TS cell medium without FGF4 and heparin, and fed every 2–3 days for 14 days as previously described (Kidder and Palmer, 2010). For cell viability analyses, trypan blue exclusion assays were performed. Briefly, shLuc and shKdm5b TS cells were re-suspended in TSC media, and 10 µl cell suspension was mixed with 10 µl trypan blue 0.4% solution. A hemacytometer and inverted microscope were used to immediately examine the cells and count the number of blue staining cells and the number of total cells. The percentage of viable cells was evaluated as follows: % viable cells=[1.00–(Number of blue cells÷Number of total cells)] ×100. Three biological replicates were evaluated.

### Lentiviral infection

shRNAs were cloned into the pGreenPuro Vector (System Biosciences, Palo Alto, USA) according to the manufacture's protocol as previously described (Kidder et al., 2013). Briefly, to generate lentiviral particles, 293T cells were co-transfected with an envelope plasmid (pLP/VSVG), packaging vector (psPAX2), and shRNA expression vector using lipofectamine 2000. Twenty-four to 48 h post transfection, the medium containing lentiviral particles was harvested and used to transduce TS cells. Twenty-four hours post transduction shLuc and shKdm5b TS cells were stably selected in the presence of 1 µg/ml puromycin.

### ChIP-Seq analysis

ChIP-Seq experiments were performed as previously described with minor modifications (Kidder et al., 2013, 2011, 2014; Kidder and Zhao, 2014). The monoclonal H3K4me3 antibody (17-614) was obtained from Millipore. The monoclonal H3K4me2 (ab32356) antibody was obtained from Abcam. Briefly, 15e6 mouse TS cells (R1) were harvested and chemically crosslinked with 1% formaldehyde (Sigma-Aldrich) for 5–10 min at 37°C and subsequently sonicated. Sonicated cell extracts equivalent to 5e6 cells were used for ChIP assays. ChIP-enriched DNA was end-repaired using the End-It DNA End-Repair kit (Epicentre, Madison, USA), followed by addition of a single A nucleotide, and ligation of custom Illumina adapters. PCR was performed using Phusion High Fidelity PCR master mix. ChIP libraries were sequenced on Illumina HiSeq platforms according to the manufacture's protocol. Sequence reads were mapped to the mouse genome (mm9) using bowtie2 (Langmead and Salzberg, 2012) with default settings. ChIP-Seq read enriched regions (peaks) were identified relative to control Input DNA using ‘Spatial Clustering for Identification of ChIP-Enriched Regions’ (SICER) software (Zang et al., 2009) with a window size setting of 200 bps, a gap setting of 400 bps, a FDR setting of 0.001. The SICER-compare function was used to compare multiple samples (FDR <0.001, fold-change >1.5). The RPBM measure (read per base per million reads) was used to quantify the density at genomic regions from ChIP-Seq datasets. We have also applied the Kolmogorov–Smirnov test to obtain *P*-value statistics and compare densities at genomic regions. Two biological replicates were performed.

### RNA-Seq analysis

Poly-A mRNA was purified using a Dynabeads mRNA purification kit. Double-stranded cDNA was generated using a Super-Script double-stranded cDNA synthesis kit (Invitrogen). cDNA was subjected to library preparation as described above. RNA-Seq libraries were sequenced on an Illumina HiSeq platform according to the manufacturer's protocol. The RPKM measure (read per kilobases of exon model per million reads) (Mortazavi et al., 2008) was used to quantify the mRNA expression level of a gene from RNA-Seq data. Differentially expressed genes were identified using edgeR [false discovery rate (FDR)<0.001; fold-change (FC)>1.5; RPKM>3] (Robinson et al., 2009). Two biological replicates were performed.
